# Association of Linezolid With Risk of Serotonin Syndrome in Patients Receiving Antidepressants

**DOI:** 10.1001/jamanetworkopen.2022.47426

**Published:** 2022-12-19

**Authors:** Anthony D. Bai, Susan McKenna, Heather Wise, Mark Loeb, Sudeep S. Gill

**Affiliations:** 1 Division of Infectious Diseases, Department of Medicine, Queen’s University, Kingston, Ontario, Canada; 2 Department of Health Research Methods, Evidence & Impact, McMaster University, Hamilton, Ontario, Canada; 3 Department of Pharmacy Services, Kingston Health Sciences Centre, Kingston, Ontario, Canada; 4 Division of Infectious Diseases, Department of Medicine, McMaster University, Hamilton, Ontario, Canada; 5 Division of Geriatric Medicine, Department of Medicine, Queen’s University, Kingston, Ontario, Canada

## Abstract

**Importance:**

Linezolid has the potential to interact with some antidepressants, leading to serotonin syndrome. However, few empirical data support warnings for patients taking antidepressants to avoid linezolid.

**Objectives:**

To examine the incidence of serotonin syndrome in patients receiving oral linezolid and how concomitant antidepressant treatment changes this risk.

**Design, Setting, and Participants:**

This population-based, retrospective cohort study used linked administrative databases at ICES to collect data from outpatients 66 years or older in Ontario, Canada, who were prescribed oral linezolid for any duration from October 1, 2014, to January 1, 2021, with follow-up to 30 days (January 31, 2021).

**Exposures:**

The use of antidepressants while receiving linezolid therapy vs no antidepressant use while receiving linezolid therapy.

**Main Outcomes and Measures:**

The primary outcome was clinically significant serotonin syndrome based on a physician diagnosis, Sternbach criteria, or the Hunter Serotonin Toxicity Criteria within 30 days of starting oral linezolid treatment. Secondary outcomes were altered mental status, hospitalization, or death within 30 days of starting linezolid treatment.

**Results:**

The study included 1134 patients (age ranges, 66-69 years for 225 patients [19.8%], 70-79 years for 473 patients [41.7%], and ≥80 years for 436 patients [38.4%]; 595 [52.5%] male) who were prescribed linezolid. Of 1134 patients, 215 (19.0%) were also taking antidepressants. Serotonin syndrome occurred in fewer than 6 patients (<0.5%). The number of serotonin syndrome cases were fewer in the antidepressant group. In a propensity score–matched cohort, the adjusted risk difference for serotonin syndrome between the antidepressant group and the no antidepressant group was −1.2% (95% CI, −2.9% to 0.5%). There were similar rates of altered mental status, hospitalization, and death between the propensity score–matched groups.

**Conclusions and Relevance:**

In this cohort study of older patients who were prescribed linezolid, serotonin syndrome occurred rarely. Concurrent antidepressants did not significantly increase the risk of serotonin syndrome. These findings suggested that linezolid is likely safe for patients receiving antidepressants. Nevertheless, prescribers should remain vigilant for this potential drug interaction.

## Key Points

**Question:**

Are patients who take antidepressants at increased risk for serotonin syndrome when receiving linezolid treatment compared with patients not taking antidepressants?

**Findings:**

In this population-based cohort study of 1134 patients who were prescribed linezolid, 215 patients (19.0%) were taking antidepressants. Serotonin syndrome occurred in less than 0.5% of patients; the adjusted risk was numerically lower in the antidepressant group, but the difference was not significantly different.

**Meaning:**

These findings suggest that antidepressants do not increase the risk of serotonin syndrome in patients receiving linezolid treatment and that linezolid is likely safe for patients receiving antidepressants.

## Teaser

This cohort study examines the incidence of serotonin syndrome among patients receiving linezolid treatment while taking an antidepressant compared with those not taking an antidepressant.

## Introduction

Linezolid is a synthetic oxazolidinone antibiotic with activity against resistant gram-positive bacteria, such as methicillin-resistant *Staphylococcus aureus* and vancomycin-resistant *Enterococcus*.^1,2^ The bioavailability of linezolid approaches 100%,^1^ making it ideal as first-line or step-down oral antibiotic therapy for bacteremia and pneumonia as well as skin and soft tissue infections.^3^

Linezolid use has been limited because of concerns of drug interactions. Linezolid can reversibly inhibit monoamine oxidase (MAO).^2^ Coadministration with antidepressants, such as nonselective MAO inhibitors, selective serotonin reuptake inhibitors (SSRIs), serotonin-norepinephrine reuptake inhibitors (SNRIs), and bupropion, may precipitate serotonin syndrome.^2^ Serotonin syndrome can present with a range of manifestations, including hyperthermia, hypertension, tachycardia, agitation, tremor, myoclonus, hyperreflexia, muscle rigidity, flushed skin, and diaphoresis.^4,5^ Severe cases may have hyperthermia and shock that are life-threatening.^4,5^

In 2020, the US Food and Drug Administration (FDA) issued a warning against linezolid use in patients taking antidepressants.^6^ Antidepressants are commonly prescribed, so many patients who needed linezolid for an infection could not receive it because of this relative contraindication.

However, data on the risk of serotonin syndrome associated with linezolid are scarce. Most of the data were case reports or case series from passive surveillance,^7,8^ which do not give any information on the incidence or how antidepressants change this risk. A review^9^ of linezolid trials showed no conclusive evidence that linezolid increased the risk of serotonin syndrome in patients already taking serotonergic medications. However, data on patients outside trials are lacking. The largest observational study^10^ to date had a sample size of 348, which included only 87 patients receiving antidepressants. In that study,^10^ concurrent antidepressant treatment conferred a relative risk (RR) of 3.00 (95% CI, 0.19-47.45) for serotonin syndrome. The small sample size likely led to imprecise estimates with a wide CI and inconclusive results. To address this knowledge gap, we conducted a retrospective cohort study using the population-based data housed at ICES in Ontario, Canada,^11^ to estimate the incidence of serotonin syndrome and how this risk changes because of concomitant antidepressant use in patients receiving linezolid treatment.

## Methods

We conducted a retrospective, population-based cohort study using the ICES databases. ICES is an independent, nonprofit research institute funded by the Ontario Ministry of Health. As a prescribed entity under Ontario’s privacy legislation, ICES is authorized to collect and use health care data for the purposes of health system analysis, evaluation, and decision support. Secure access to these data is governed by policies and procedures that are approved by the Information and Privacy Commissioner of Ontario.^11^ The ICES data policy requires that research outputs and reports must not contain information that identifies an individual or could foreseeably be used to reidentify an individual. Therefore, all cells containing or revealing 5 individuals or fewer were suppressed to comply with this policy. This study was approved by the Queen’s University Health Sciences & Affiliated Teaching Hospitals Research Ethics Board. This study was reported as per the Strengthening the Reporting of Observational Studies in Epidemiology (STROBE) and Reporting of Studies Conducted Using Observational Routinely-Collected Data (RECORD) reporting guidelines (eTable 1 in Supplement 1).^12^

### Population

The ICES databases included the Ontario Drug Benefit (ODB) database, which captured outpatient prescription medications dispensed to people 65 years or older in Ontario, Canada. Oral linezolid was originally listed on the formulary with restrictions and then added to the ODB database starting in October 2014.^13^ Therefore, this study included adults 66 years or older in Ontario who were dispensed oral linezolid for any duration between October 1, 2014, and January 1, 2021. This was a convenient sample size based on the study date cutoff. Patients were followed up for 30 days (January 31, 2021).

### Data Collection

The ODB data were linked to the following databases at a person level. Census data included demographic characteristics and vital statistics. The Canadian Institute for Health Information discharge abstract database, National Ambulatory Care Reporting System, and Ontario Health Insurance Plan database had information on diagnoses and health care use, including ambulatory care visits, emergency department (ED) visits, and hospitalizations. Finally, Ontario Laboratories Information Systems included blood work results. All patients had complete linked data.

The following information was collected for each patient: (1) demographic characteristics, including age, sex, and rural or urban home address; (2) comorbidities as determined by the Charlson Comorbidity Index^14^; (3) psychiatric diagnoses, such as substance use disorder; (4) blood work, including estimated glomerular filtration rate based on baseline serum creatinine level^15^; and (5) linezolid dose, frequency, and treatment start date and stop dates. The investigators had access to the deidentified database that was prepared by the ICES team based on the eligibility criteria on a secure online server for analysis. Data cleaning was done by data checking and removing data outside the relevant time frame for the study.

### Exposure

The exposure of interest was concomitant oral antidepressants while taking linezolid that were known to predispose patients to serotonin syndrome as per the FDA warnings.^6^ These include (1) SSRIs: paroxetine, fluvoxamine, fluoxetine, sertraline, citalopram, and escitalopram; (2) SNRIs: venlafaxine, desvenlafaxine, and duloxetine; (3) tricyclic antidepressants: clomipramine, amitriptyline, desipramine, imipramine, nortriptyline, protriptyline, doxepin, and trimipramine; (4) MAO inhibitors: isocarboxazid, phenelzine, selegiline, tranylcypromine, and moclobemide; (5) norepinephrine and dopamine reuptake inhibitors: bupropion; and (6) other: trazodone, mirtazapine, buspirone, amoxapine, maprotiline, and nefazodone.

We also collected data on other concomitant prescribed serotonergic medications that may increase the risk of serotonin syndrome^4,7,8^ and were covered by the ODB. These include lithium, serotonin 3 receptor antagonists (dolasetron, granisetron, ondansetron, and palonosetron), metoclopramide, methylphenidate, dexmethylphenidate, fentanyl, meperidine, methadone, oxycodone, tramadol, triptans (almotriptan, eletriptan, frovatriptan, naratriptan, rizatriptan, sumatriptan, and zolmitriptan), ergot derivatives (dihydroergotamine, ergotamine, and methylergonovine), dextromethorphan, chlorpheniramine, cyclobenzaprine, carbamazepine, valproic acid, tryptophan, dextroamphetamine, rasagiline, and ritonavir.

### Outcomes

The primary outcome was clinically significant serotonin syndrome requiring an ambulatory care visit, ED visit, or hospitalization. The discharge diagnoses from ambulatory care visits, ED visits, and hospitalization were captured by the *International Statistical Classification of Diseases and Related Health Problems, Tenth Revision* (*ICD-10*) codes. On the basis of the *ICD-10* codes, serotonin syndrome was defined as any of the following: diagnosis of drug-induced serotonin syndrome or toxic effect related to serotonergic drugs by the physician, diagnosis based on Sternbach criteria,^16^ or diagnosis based on the Hunter Serotonin Toxicity Criteria (eTable 2 in Supplement 1).^17^ Serotonin syndrome must have occurred within 30 days of starting linezolid treatment. This time frame was considered reasonable because linezolid is typically prescribed for no more than 14 days and should be cleared from the system in 2 days after discontinued use given its half-life of 5 to 7 hours.^1,18^

Secondary outcomes were chosen to describe potential consequences of clinically significant serotonin syndrome that may not be captured by the criteria for serotonin syndrome as described herein. All secondary outcomes were defined to be within 30 days of starting linezolid treatment: (1) ED visit or hospitalization for acute altered mental status change or confusion, (2) hospitalization for any cause, or (3) death from any cause.

### Statistical Analysis

Descriptive analysis included means (SDs) or medians (IQRs) for continuous variables. Numbers (percentages) were used to describe categorical variables. Primary and secondary outcomes were compared between the antidepressant and no antidepressant groups using the Fisher exact test and absolute risk differences with 95% CI as per the Newcombe method.^19^ Risk difference was calculated as the risk in the antidepressant group minus the risk in the no antidepressant group. There was no loss to follow-up because data for all outcomes were captured by the administrative databases.

To address potential bias, a propensity score for antidepressant use was estimated using a logistic regression of the following patient baseline characteristics chosen a priori: age, sex, rural home address, Charlson Comorbidity Index, estimated glomerular filtration rate, history of substance use disorder, and days of linezolid use and other serotonergic medications. Age in years was categorized into 66 to 69, 70 to 79, and 80 or older. Patients taking antidepressants were matched in a 1:1 ratio to patients not taking antidepressants using nearest neighbor matching with a specified caliper width of 0.1 times the SD of the logit of propensity scores. The 2 propensity score–matched groups were then compared in terms of the primary and secondary outcomes using a complete case analysis. To account for the matched nature of samples,^20^ statistical significance and 95% CIs for risk difference were estimated using the exact McNemar test and variance of the risk difference as described by Agresti and Min,^21^ respectively.

All reported CIs were 2-sided 95% intervals, and all tests were 2-sided with a *P* < .05 significance level. All analyses were performed using statistical software R, versions 3.3.0 and 4.1.2 (R Foundation for Statistical Computing). The statistical packages DescTools, MatchIt, and exact 2 × 2 were used for risk difference CI estimation, propensity score matching, and exact McNemar test, respectively.^22,23,24^

## Results

### Patient Characteristics

The study included 1134 patients (age ranges, 66-69 years for 225 patients [19.8%], 70-79 years for 473 patients [41.7%], and ≥80 years for 436 patients [38.4%]; 595 [52.5%] male and 539 [47.5%] female) who were prescribed linezolid. All eligible patients were included in the study, linked by the databases, and completed follow-up to 30 days. All linezolid prescriptions were for 600-mg tablets to be taken twice daily. Of the 1134 patients, 215 (19.0%) were taking antidepressants. In terms of antidepressant classes, 103 patients (47.9%) were taking an SSRI, 36 (16.7%) were taking an SNRI, 15 (7.0%) were taking a tricyclic antidepressant, 7 (3.3%) were taking a norepinephrine and dopamine reuptake inhibitor, and no patients were taking an MAO inhibitor. eTable 3 in Supplement 1 describes the proportions of patients by antidepressant type. For the 215 patients taking antidepressants, the median time of overlap for linezolid and antidepressant was 7 days (IQR, 5-10 days). In 142 patients (66%), the antidepressant was taken during the entire linezolid course. In 197 patients (91.6%), antidepressant use overlapped with linezolid therapy for 3 days or more.

Baseline characteristics for patients taking and not taking antidepressants are given in Table 1. Of note, 19 patients (8.8%) in the antidepressant group and 47 patients (5.1%) in the no antidepressant group were also taking other serotonergic medications. The proportions of patients in each group by type of other serotonergic medications are given in eTable 4 in Supplement 1.

**Table 1.** Baseline Characteristics of the Study Patients^a^

Characteristic	Antidepressant group (n = 215)	No antidepressant group (n = 919)	Standardized difference
Age group, y			
66-69	48 (22.3)	177 (19.3)	0.0756
70-79	83 (38.6)	390 (42.4)	0.0781
≥80	84 (39.1)	352 (38.3)	0.0158
Sex			
Female	117 (54.4)	422 (45.9)	0.1706
Male	98 (45.6)	497 (43.8)	0.1706
Rural home address	23 (10.7)	105 (11.4)	0.0216
Charlson Comorbidity Index, mean (SD)^b^	2.3 (2.1)	2.0 (2.1)	0.1200
eGFR, mean (SD), mL/min/1.73 m^2^^c^	70.7 (28.3)	69.6 (27.2)	0.0393
History of substance use disorder	≤5	≤5	0.0221
Days of linezolid treatment, mean (SD)	11.7 (7.2)	10.3 (6.2)	0.2089
No. of antidepressants, mean (SD)	1.2 (0.5)	0	3.8008
Taking other serotonergic medication(s)	19 (8.8)	47 (5.1)	0.1465

Abbreviation: eGFR, estimated glomerular filtration rate.

^a^ Data are presented as number (percentage) of patients unless otherwise indicated.

^b^ Data available for 175 patients in the antidepressant group and 760 patients in the no antidepressant group.

^c^ Data available for 208 patients in the antidepressant group and 897 patients in the no antidepressant group.

### Serotonin Syndrome

Serotonin syndrome occurred in fewer than 6 patients (<0.5%) in total. The exact numbers were not reported as per ICES data policy on potentially identifiable patient information. Based on fewer than 6 events, the possible risk difference for serotonin syndrome ranged from −0.5% to 2.3%. The number of serotonin syndrome cases were fewer in the antidepressant group. Secondary outcomes are described in Table 2.

**Table 2.** Secondary Outcomes Within 30 Days of Starting Linezolid Treatment

Secondary outcome	No. (%) of patients		Risk difference, % (95% CI)^a^	*P* value
	Antidepressant group (n = 215)	No antidepressant group (n = 919)		
Altered mental status or confusion	25 (11.6)	63 (6.9)	4.8 (0.7 to 10.1)	.02
Hospitalization	92 (42.8)	398 (43.3)	−0.5 (−7.7 to 6.9)	.93
Death from any cause	16 (7.4)	44 (4.8)	2.7 (−0.6 to 7.1)	.13

^a^ Risk difference was calculated as risk in the antidepressant group minus risk in the no antidepressant group.

### Propensity Score Matching

Using propensity scores, 166 patients in the antidepressant group were matched to 166 patients in the no antidepressant group. The maximum standardized difference of matched variables was 0.0845 (Table 3), which suggested good balance on the measured baseline characteristics. In this propensity score–matched cohort, the risk of serotonin syndrome was lower in the antidepressant group, with an adjusted risk difference of −1.2% (95% CI, −2.9% to 0.5%; *P* = .50). Within this 95% CI, the worst-case scenario would be a 0.5% increase in the risk of serotonin syndrome due to antidepressants, which is equivalent to a number needed to harm of 200. There were similar rates of altered mental status or confusion, hospitalization, and death within 30 days between the 2 propensity score–matched groups (Table 4).

**Table 3.** Baseline Characteristics in Propensity Score–Matched Cohort^a^

Characteristic	Antidepressant group (n = 166)	No antidepressant group (n = 166)	Standardized difference
Age group, y			
66-69	39 (23.5)	37 (22.3)	0.0287
70-79	68 (41.0)	65 (39.2)	0.0369
≥80	59 (35.5)	64 (38.6)	0.0624
Sex			
Female	84 (50.6)	91 (54.8)	0.0845
Male	82 (49.4)	75 (45.2)	0.0845
Rural home address	21 (12.7)	18 (10.8)	0.0562
Charlson Comorbidity Index, mean (SD)	2.2 (1.9)	2.1 (2.1)	0.0358
eGFR, mean (SD), mL/min/1.73 m^2^	70.4 (29.0)	70.4 (27.1)	0.0003
History of substance use disorder	0	0	0
Days of linezolid treatment, mean (SD)	10.6 (6.4)	10.7 (6.3)	0.0161
Taking other serotonergic medication(s)	11 (6.6)	8 (4.8)	0.0779

Abbreviation: eGFR, estimated glomerular filtration rate.

^a^ Data are presented as number (percentage) of patients unless otherwise indicated. Listwise deletion was used, so no data were missing in the propensity score–matched cohort.

**Table 4.** Secondary Outcomes Within 30 Days of Starting Linezolid Treatment in Propensity Score–Matched Cohort

Secondary outcome	No. (%) of patients		Risk difference, % (95% CI)^a^	*P* value^b^
	Antidepressant group (n = 166)	No antidepressant group (n = 166)		
Altered mental status or confusion	20 (12.1)	19 (11.5)	0.6 (−6.2 to 7.4)	>.99
Hospitalization	82 (49.4)	85 (51.2)	−1.8 (−11.9 to 8.3)	.82
Death from any cause	14 (8.4)	10 (6.0)	2.4 (−3.4 to 8.2)	.54

^a^ Risk difference was calculated as risk in the antidepressant group minus risk in the no antidepressant group.

^b^ *P* value by the McNemar exact test.

## Discussion

In this large, population-based, retrospective cohort study of 1134 outpatients who received oral linezolid, serotonin syndrome was rare and occurred in less than 0.5% of patients. After matching by propensity score, the risk difference for serotonin syndrome was −1.2% (95% CI, −2.9% to 0.5%) when compared between the antidepressant and no antidepressant groups. Similarly, there were no significant differences in secondary outcomes related to consequences from serotonin syndrome, including altered mental status or confusion, hospitalization, and death. Thus, antidepressants did not significantly increase the risk of serotonin syndrome while receiving linezolid treatment.

In a secondary analysis^9^ of 20 randomized clinical trials that compared linezolid with a comparator agent in patients who were taking at least 1 serotonergic agent, serotonin syndrome occurred in 3 of 2208 patients (0.14%) in the linezolid group and 1 of 2057 patients (0.05%) in the comparator group (RR, 2.79; 95% CI, 0.29-26.85). The authors concluded that the potential risk of serotonin syndrome in patients already taking a serotonergic agent was low,^9^ which is consistent with our study finding. Our study adds to this previous study^9^ by comparing serotonin syndrome risk associated with linezolid with a focus on antidepressants while adjusting for demographic characteristics, comorbidities, kidney function, substance use disorder, and medications. Moreover, our study described data on an aging population in a community setting with multiple comorbidities, which reflects the population being treated with linezolid in clinical practice that is different from trials. For example, more than 70% of the population were younger than 65 years in the trial population,^9^ whereas all patients in our study were older than 65 years. To our knowledge, our study is the largest observational study to date on the risk of serotonin syndrome associated with linezolid use outside the context of clinical trials. The next largest study^10^ was a single-center observational study of 348 inpatients taking linezolid. In that study,^10^ serotonin syndrome was diagnosed in 1.1% of patients taking an SSRI or SNRI vs 0.4% of patients not taking an SSRI or SNRI (RR, 3.00; 95% CI, 0.19-47.45). With more than 3 times the sample size, our study allowed for a more precise estimate and presented absolute risk differences, which may be easier to interpret and less misleading than RR given the very low event rate.^25^

The evidence for rare and serious adverse events associated with antibiotics often comes from retrospective, population-based observational studies, such as our study. This evidence has limitations due to the nature of retrospective observational studies. As well, such studies are not efficient because they often focus on a particular adverse event. Future research should move beyond observational studies to phase 4 studies, which would prospectively monitor for all types of adverse events. While waiting for higher-quality evidence, our study adds to the existing evidence for the safety of linezolid even in the context of concomitant antidepressants. Based on the existing evidence, clinicians should be reassured that it appears safe to prescribe oral linezolid to patients taking antidepressants, especially if there are limited antibiotic options or alternative antibiotic options would be inferior.

### Strengths and Limitations

Our study has several strengths. First, it is a large cohort study with more than 1000 patients, which allows for more precise estimates. Second, the study population consisted of all elderly patients with multiple comorbidities and medications, which is the most vulnerable population at the highest risk for medication adverse events that is representative of the typical patients being prescribed linezolid in clinical practice. Third, multiple linked administrative databases allowed for detailed and comprehensive information on demographic characteristics, socioeconomic status, comorbidities, and blood work results, which were then adjusted using propensity scores when comparing the primary and secondary outcomes between the 2 groups.

Several limitations merit mentioning. First, serotonin syndrome was diagnosed retrospectively based on a constellation of diagnosis codes, which did not match perfectly with the diagnostic criteria. However, this is the most common method used in previous studies.^8,9^ We used multiple definitions for serotonin syndrome (physician diagnosis, Sternbach criteria, and the Hunter Serotonin Toxicity Criteria) to be inclusive. Physician diagnosis complemented the diagnostic criteria because the diagnosis codes for individual symptoms were likely omitted when an overarching physician diagnosis was entered into the hospital records. Still, our serotonin syndrome criteria may not be sensitive enough to capture all cases. This limitation is addressed by the secondary outcomes that included altered mental status or confusion, hospitalization, and death. Any clinically significant serotonin syndrome likely resulted in 1 of these secondary outcomes. It is reassuring that antidepressants did not significantly increase the risk of serotonin syndrome or the secondary outcomes after adjustment by propensity score matching. The observed risk of serotonin syndrome at less than 0.5% in our study is similar to the estimated incidence of 0.14% in clinical trials^9^ and 0.6% in another observational study,^10^ which suggests that we did not miss a significant number of cases.

Second, the small number of events limited the possible analyses and precision of the estimates. For example, it was not possible to perform a multivariable logistic regression model because of the low number of events. As well, odds ratios or RRs may be extreme and misleading because of low event rates. We were able to adjust for patient baseline characteristics using propensity scores, which is an appropriate method to adjust for confounders independent of event rate.^26^ Propensity score matching also allows for calculation of risk differences,^20^ which is related to the number needed to harm and thus easier to interpret for clinicians. The low number of events is still useful in the context of a large sample size. Fewer than 6 cases of serotonin syndrome among more than 1000 patients with a risk difference CI limit of an excess risk of 0.5% was reassuring in that serotonin syndrome was rare and antidepressants did not significantly add to this risk.

Third, only prescribed and funded medications were captured by the ODB. Over-the-counter medications not funded by the ODB would be missed, but most clinically important serotonergic medications were prescription medications. The database also did not account for medication adherence. However, this limitation would reflect a population-level intent-to-treat effect of prescribing linezolid to a patient who was also prescribed antidepressants. It would be unlikely for prescribers to recommend abruptly stopping use of an antidepressant temporarily while taking linezolid because of the risk of antidepressant discontinuation syndrome.

Of note, no patient was taking an MAO inhibitor antidepressant in our study, which has a very high risk of serotonin syndrome. This lack of MAO inhibitor use reflects the current practice in which MAO inhibitor antidepressants are rarely used. Therefore, this study may not be generalized to patients taking MAO inhibitor antidepressants.

## Conclusions

In this cohort study, concomitant antidepressants did not significantly increase the risk of serotonin syndrome in patients taking linezolid. Within the 95% CI, the worst-case scenario would be a 0.5% increase in the risk of serotonin syndrome due to antidepressants, which would be a number needed to harm of 200. As such, it is likely safe to prescribe linezolid in patients taking antidepressants, and antidepressants should not be an absolute contraindication for linezolid.

## Notes

Supplement 1. **eTable 1.** The RECORD Statement—Checklist of Items, Extended From the STROBE Statement, That Should Be Reported in Observational Studies Using Routinely Collected Health Data **eTable 2.** Definition of Serotonin Syndrome Based on *ICD-10* Codes **eTable 3.** Proportion of Patients on Different Types of Antidepressants **eTable 4.** Proportion of Patients on Other Serotonergic Medications

Supplement 2.
Data Sharing Statement
