# Supplementary material for: Association of Linezolid With Risk of Serotonin Syndrome in Patients Receiving Antidepressants
Source: JAMA Netw Open. 2022 Dec 19;5(12):e2247426. doi: 10.1001/jamanetworkopen.2022.47426 (PMC9856528; doi:10.1001/jamanetworkopen.2022.47426)
Supplement: Supplement 1. — eTable 1. The RECORD Statement—Checklist of Items, Extended From the STROBE Statement, That Should Be Reported in Observational Studies Using Routinely Collected Health Data eTable 2. Definition of Serotonin Syndrome Based on ICD-10 Codes eTable 3. Proportion of Patients on Different Types of Antidepressants eTable 4. Proportion of Patients on Other Serotonergic Medications [file jamanetwopen-e2247426-s001.pdf]

Supplementary Online Content

Bai AD, McKenna S, Wise H, Loeb M, Gill SS. Association of linezolid and risk of serotonin syndrome in patients receiving antidepressants. *JAMA Netw Open*. 2022;5(12):e2247426. doi:10.1001/jamanetworkopen.2022.47426

eTable 1. The RECORD Statement—Checklist of Items, Extended From the STROBE Statement, That Should Be Reported in Observational Studies Using Routinely Collected Health Data
eTable 2. Definition of Serotonin Syndrome Based on *ICD-10* Codes
eTable 3. Proportion of Patients on Different Types of Antidepressants
eTable 4. Proportion of Patients on Other Serotonergic Medications

This supplementary material has been provided by the authors to give readers additional information about their work.

© 2022 Bai AD et al. *JAMA Network Open.*

eTable 1. The RECORD Statement—Checklist of Items, Extended From the STROBE Statement, That Should Be Reported in Observational Studies Using Routinely Collected Health Data

	Item No.	STROBE items	Location in manuscript where items are reported	RECORD items	Location in manuscript where items are reported
**Title and abstract**					
	1	(a) Indicate the study's design with a commonly used term in the title or the abstract<br>(b) Provide in the abstract an informative and balanced summary of what was done and what was found	1<br><br><br>1	RECORD 1.1: The type of data used should be specified in the title or abstract. When possible, the name of the databases used should be included.<br><br>RECORD 1.2: If applicable, the geographic region and timeframe within which the study took place should be reported in the title or abstract.<br><br>RECORD 1.3: If linkage between databases was conducted for the study, this should be clearly stated in the title or abstract.	1<br><br><br><br><br>1<br><br><br><br><br>1
**Introduction**					
Background rationale	2	Explain the scientific background and rationale for the investigation being reported	2		
Objectives	3	State specific objectives, including any prespecified hypotheses	2		
**Methods**					
Study Design	4	Present key elements of study design early in the paper	2		
Setting	5	Describe the setting, locations, and relevant dates, including periods of recruitment, exposure, follow-up, and data collection	2		
Participants	6	*(a) Cohort study* - Give the eligibility criteria, and the sources and methods of selection	2	RECORD 6.1: The methods of study population selection (such as codes or algorithms used to identify subjects) should	2

© 2022 Bai AD et al. *JAMA Network Open.*

		of participants. Describe methods of follow-up<br>*Case-control study* - Give the eligibility criteria, and the sources and methods of case ascertainment and control selection. Give the rationale for the choice of cases and controls<br>*Cross-sectional study* - Give the eligibility criteria, and the sources and methods of selection of participants<br><br>*(b) Cohort study* - For matched studies, give matching criteria and number of exposed and unexposed<br>*Case-control study* - For matched studies, give matching criteria and the number of controls per case	 <br><br><br><br>N/A	be listed in detail. If this is not possible, an explanation should be provided.<br><br>RECORD 6.2: Any validation studies of the codes or algorithms used to select the population should be referenced. If validation was conducted for this study and not published elsewhere, detailed methods and results should be provided.<br><br>RECORD 6.3: If the study involved linkage of databases, consider use of a flow diagram or other graphical display to demonstrate the data linkage process, including the number of individuals with linked data at each stage.	 <br>N/A<br><br><br>3 for description<br>Figure not used
Variables	7	Clearly define all outcomes, exposures, predictors, potential confounders, and effect modifiers. Give diagnostic criteria, if applicable.	3-4	RECORD 7.1: A complete list of codes and algorithms used to classify exposures, outcomes, confounders, and effect modifiers should be provided. If these cannot be reported, an explanation should be provided.	3-4<br>Supplement Table e2
Data sources/ measurement	8	For each variable of interest, give sources of data and details of methods of assessment (measurement).<br>Describe comparability of assessment methods if there is more than one group	3-4		
Bias	9	Describe any efforts to address potential sources of bias	4		
Study size	10	Explain how the study size was arrived at	3		

© 2022 Bai AD et al. *JAMA Network Open.*

Quantitative variables	11	Explain how quantitative variables were handled in the analyses. If applicable, describe which groupings were chosen, and why	4		
Statistical methods	12	(a) Describe all statistical methods, including those used to control for confounding<br>(b) Describe any methods used to examine subgroups and interactions<br>(c) Explain how missing data were addressed<br>(d) *Cohort study* - If applicable, explain how loss to follow-up was addressed<br>*Case-control study* - If applicable, explain how matching of cases and controls was addressed<br>*Cross-sectional study* - If applicable, describe analytical methods taking account of sampling strategy<br>(e) Describe any sensitivity analyses	4<br>N/A<br>4<br>4<br>N/A		
Data access and cleaning methods		..		RECORD 12.1: Authors should describe the extent to which the investigators had access to the database population used to create the study population.<br><br>RECORD 12.2: Authors should provide information on the data cleaning methods used in the study.	3<br><br>3
Linkage		..		RECORD 12.3: State whether the study included person-level, institutional-level, or other data linkage across two or more databases. The methods of linkage and	3

© 2022 Bai AD et al. *JAMA Network Open.*

				methods of linkage quality evaluation should be provided.	
**Results**					
Participants	13	(a) Report the numbers of individuals at each stage of the study (*e.g.*, numbers potentially eligible, examined for eligibility, confirmed eligible, included in the study, completing follow-up, and analysed)<br>(b) Give reasons for non-participation at each stage.<br>(c) Consider use of a flow diagram	4<br><br>4<br><br>Not used	RECORD 13.1: Describe in detail the selection of the persons included in the study (*i.e.*, study population selection) including filtering based on data quality, data availability and linkage. The selection of included persons can be described in the text and/or by means of the study flow diagram.	4
Descriptive data	14	(a) Give characteristics of study participants (*e.g.*, demographic, clinical, social) and information on exposures and potential confounders<br>(b) Indicate the number of participants with missing data for each variable of interest<br>(c) *Cohort study* - summarise follow-up time (*e.g.*, average and total amount)	Table 1<br><br>Table 1<br><br>4		
Outcome data	15	*Cohort study* - Report numbers of outcome events or summary measures over time<br>*Case-control study* - Report numbers in each exposure category, or summary measures of exposure<br>*Cross-sectional study* - Report numbers of outcome events or summary measures	4<br>Table 2		
Main results	16	(a) Give unadjusted estimates and, if applicable, confounder-adjusted estimates and their	4-5<br>Table 2<br>Table 4		

© 2022 Bai AD et al. *JAMA Network Open.*

		precision (e.g., 95% confidence interval). Make clear which confounders were adjusted for and why they were included<br>(b) Report category boundaries when continuous variables were categorized<br>(c) If relevant, consider translating estimates of relative risk into absolute risk for a meaningful time period	Table 1 and 3<br><br>4-5<br>Table 2<br>Table 4		
Other analyses	17	Report other analyses done—e.g., analyses of subgroups and interactions, and sensitivity analyses	N/A		
**Discussion**					
Key results	18	Summarise key results with reference to study objectives	5		
Limitations	19	Discuss limitations of the study, taking into account sources of potential bias or imprecision. Discuss both direction and magnitude of any potential bias	7	RECORD 19.1: Discuss the implications of using data that were not created or collected to answer the specific research question(s). Include discussion of misclassification bias, unmeasured confounding, missing data, and changing eligibility over time, as they pertain to the study being reported.	7
Interpretation	20	Give a cautious overall interpretation of results considering objectives, limitations, multiplicity of analyses, results from similar studies, and other relevant evidence	5-6		
Generalisability	21	Discuss the generalisability (external validity) of the study results	7-8		
**Other Information**					
Funding	22	Give the source of funding and the role of the funders for the	8		

© 2022 Bai AD et al. *JAMA Network Open.*

		present study and, if applicable, for the original study on which the present article is based			
Accessibility of protocol, raw data, and programming code		..	8	RECORD 22.1: Authors should provide information on how to access any supplemental information such as the study protocol, raw data, or programming code.	8

*Reference: Benchimol EI, Smeeth L, Guttmann A, Harron K, Moher D, Petersen I, Sørensen HT, von Elm E, Langan SM, the RECORD Working Committee. The REporting of studies Conducted using Observational Routinely-collected health Data (RECORD) Statement. *PLoS Medicine* 2015; in press.

*Checklist is protected under Creative Commons Attribution (CC BY) license.

eTable 2. Definition of Serotonin Syndrome Based on *ICD-10* codes

	ICD-10 codes[a]
Physician diagnosis	T43, T44<br>F19
Mental status change	F03, F05, F19<br>G45.9<br>R41.0, R41.8, R40.224
Agitation	R45.0, R45.1<br>F43.0, F43.8
Myoclonus or spontaneous clonus	G25.3<br>R25.2
Hyperreflexia	R29.2
Diaphoresis	R61
Shivering	R68.83
Tremor	G25<br>R25.1
Diarrhea	R19.4, R19.7<br>K52.9, K58<br>A08, A09
Incoordination	R27<br>G11, G32.81
Fever	R50<br>A68
Ocular clonus	H55
Hypertonia	G24

[a]Codes without decimal numbers specified mean that all possible decimal numbers that begin with the same first 3 characters are all included

Sternbach criteria (reference: Sternbach H. The serotonin syndrome. *Am J Psychiatry*. 1991;148:705-13.) based on >=3 of the following: mental status change, agitation, myoclonus, hyperreflexia, diaphoresis, shivering, tremor, diarrhea, incoordination, fever

Hunter's criteria (reference: Dunkley EJ, Isbister GK, Sibbritt D, Dawson AH, Whyte IM. The Hunter Serotonin Toxicity Criteria: simple and accurate diagnostic decision rules for serotonin toxicity. QJM. 2003 Sep;96(9):635-42. doi: 10.1093/qjmed/hcg109.) based on any of the following:

- (Inducible or ocular clonus) plus (agitation or diaphoresis)
- Tremor plus hyperreflexia
- Hypertonia plus fever plus (ocular or inducible clonus)
- Spontaneous clonus

© 2022 Bai AD et al. *JAMA Network Open.*

eTable 3. Proportion of Patients on Different Types of Antidepressants

	Patients on antidepressants (N=215)
Selective serotonin reuptake inhibitor (SSRI)	
Paroxetine	8 (3.7%)
Sertraline	25 (11.6%)
Citalopram	65 (30.2%)
Escitalopram	0 (0%)
Fluvoxamine	≤5
Fluoxetine	≤5
Serotonin and norepinephrine reuptake inhibitor (SNRI)	
Venlafaxine	11 (5.1%)
Desvenlafaxine	0 (0%)
Duloxetine	25 (11.6%)
Tricyclic antidepressants (TCA)	
Clomipramine	0 (0%)
Amitriptyline	13 (6.1%)
Desipramine	0 (0%)
Imipramine	≤5
Nortriptyline	0 (0%)
Protriptyline	0 (0%)
Doxepin	≤5
Trimipramine	0 (0%)
Monoamine oxidase inhibitors (MAOI)	
Isocarboxazid	0 (0%)
Phenelzine	0 (0%)
Selegiline	0 (0%)
Tranylcypromine	0 (0%)
Moclobemide	0 (0%)
Norepinephrine and dopamine reuptake inhibitor (NDRI)	
Bupropion	7 (3.3%)
Other	
Trazodone	76 (35.4%)
Mirtazapine	22 (10.2%)
Amoxapine	0 (0%)
Maprotiline	0 (0%)
Nefazodone	0 (0%)

© 2022 Bai AD et al. *JAMA Network Open.*

eTable 4. Proportion of patients on other serotonergic medications

	Antidepressant (N=215)	No antidepressant (N=919)
Lithium	0 (0%)	0 (0%)
Dolasetron	0 (0%)	0 (0%)
Granisetron	0 (0%)	0 (0%)
Ondansetron	≤5	13 (1.4%)
Palonosetron	0 (0%)	0 (0%)
Metoclopramide	8 (3.7%)	11 (1.2%)
Alomitriptan	0 (0%)	0 (0%)
Eletriptan	0 (0%)	0 (0%)
Frovatriptan	0 (0%)	0 (0%)
Naratriptan	0 (0%)	0 (0%)
Rizatriptan	0 (0%)	0 (0%)
Sumatriptan	0 (0%)	0 (0%)
Zolmitriptan	0 (0%)	0 (0%)
Dihydroergotamine	0 (0%)	0 (0%)
Ergotamine	0 (0%)	0 (0%)
Methylergonovine	0 (0%)	0 (0%)
Fentanyl	≤5	7 (0.8%)
Meperidine	0 (0%)	0 (0%)
Methadone	0 (0%)	0 (0%)
Oxycodone	≤5	≤5
Tramadol	0 (0%)	0 (0%)
Dextromethorphan	0 (0%)	0 (0%)
Chlorpheniramine	0 (0%)	0 (0%)
Cyclobenzaprine	0 (0%)	0 (0%)
Carbamazepine	≤5	0 (0%)
Valproic acid		≤5
Tryptophan	≤5	11 (1.2%)
Methylphenidate	0 (0%)	0 (0%)
Dexmethylphenidate	0 (0%)	0 (0%)
Dextroamphetamine	0 (0%)	0 (0%)
Rasagiline	≤5	0 (0%)
Ritonavir	0 (0%)	0 (0%)

© 2022 Bai AD et al. *JAMA Network Open.*
