# Supplementary material for: Association of Linezolid With Risk of Serotonin Syndrome in Patients Receiving Antidepressants
Source: JAMA Netw Open. 2022 Dec 19;5(12):e2247426. doi: 10.1001/jamanetworkopen.2022.47426 (PMC9856528; doi:10.1001/jamanetworkopen.2022.47426)
Supplement: Supplement 2. — Data Sharing Statement [file jamanetwopen-e2247426-s002.pdf]

Data Sharing Statement

Bai. Association of Linezolid With Risk of Serotonin Syndrome in Patients Receiving Antidepressants. *JAMA Netw Open*. Published December 19, 2022. doi:10.1001/jamanetworkopen.2022.47426

Data

Data available: The data set and analysis codes for this study is held securely in coded form at the Institute for Clinical Evaluative Sciences (ICES). While data sharing agreements prohibit ICES from making the data set publicly available, access may be granted to those who meet prespecified criteria for confidential access, available at http://www.ices.on.ca/DAS. The full data set creation plan and underlying analytic code are available from the authors on request, understanding that the computer programs may rely on coding templates or macros that are unique to ICES and are therefore either inaccessible or may require modification.
